# Occupational radiation exposure to nursing staff during cardiovascular fluoroscopic procedures: A review of the literature

**DOI:** 10.1002/acm2.12461

**Published:** 2018-10-08

**Authors:** Kelly Wilson‐Stewart, Madeleine Shanahan, Davide Fontanarosa, Rob Davidson

**Affiliations:** ^1^ School of Clinical Sciences Queensland University of Technology Brisbane Qld Australia; ^2^ Cardiovascular Suites Greenslopes Private Hospital Brisbane Qld Australia; ^3^ Faculty of Health University of Canberra Canberra ACT Australia; ^4^ Institute of Health and Biomedical Innovation Queensland University of Technology Brisbane Qld Australia

**Keywords:** fluoroscopy, nursing, occupational exposure, radiation exposure, systematic review

## Abstract

Fluoroscopy is a method used to provide real time x‐ray imaging of the body during medical procedures to assist with medical diagnosis and treatment. Recent technological advances have seen an increase in the number of fluoroscopic examinations being performed. Nurses are an integral part of the team conducting fluoroscopic investigations and are often located close to the patient resulting in an occupational exposure to radiation. The purpose of this review was to examine recent literature which investigates occupational exposure received by nursing staff during cardiovascular fluoroscopic procedures. Articles published between 2011 and 2017 have been searched and comprehensively reviewed on the referenced medical search engines. Twenty‐four relevant studies were identified among which seventeen investigated nursing dose comparative to operator dose. Seven researched the effectiveness of interventions in reducing occupational exposure to nursing staff. While doctors remain at the highest risk of exposure during procedures, evidence suggests that nursing staff may be at risk of exceeding recommended dose limits in some circumstances. There is also evidence of inconsistent use of personal protection such as lead glasses and skull caps by nursing staff to minimize radiation exposure. Conclusions: The review has highlighted a lack of published literature focussing on dose to nurses. There is a need for future research in this area to inform nursing staff of factors which may contribute to high occupational doses and of methods for minimizing the risk of exposure, particularly regarding the importance of utilizing radiation protective equipment.

**Abbreviations**
ALARA: as low as reasonably achievable
CV: cardiovascular
DAP: Dose Area Product
DSA: digital subtraction angiography
EP: electrophysiology
EVAR: endovascular aneurysm repair
H_p_(0.07): calibration of a dose meter to detect the personal dose equivalent at 0.07 mm depth in tissue
H_p_(3): calibration of a dose meter to detect the personal dose equivalent at 3 mm depth in tissue
H_p_(10): calibration of a dose meter to detect the personal dose equivalent at 10 mm depth in tissue
IC: interventional cardiology
ICRP: International Commission on Radiological Protection
INR: interventional neuro‐radiology
IR: interventional radiology
KAP: kerma area product
mSv: milliSievert
NR: neuroradiology
PDM: personal dose meter

## 1 INTRODUCTION

Fluoroscopy is a method used to provide real time imaging of the body during medical procedures. It utilizes x‐rays which pass through the patient to visualize internal structures. Historically x‐ray fluoroscopy was primarily used for diagnosis, but recent advances in both imaging and procedural equipment have led to considerable growth in the range of fluoroscopically guided procedures, particularly in the field of interventional cardiology, (IC) and vascular intervention.^1^, ^2^, ^3^ Interventional cardiovascular (CV) cases are often less costly than surgery and allow medical intervention to be conducted in a minimally invasive way, reducing the risk to the patient.^4^

Although very useful for imaging, ionizing radiation may result in several detrimental effects to those exposed, including cellular damage, malignancies, and cataracts.^5^, ^6^, ^7^, ^8^ The greatest risk of occupational exposure occurs when the primary x‐ray beam strikes the patient's skin and scatters, a portion of the x‐ray photons are absorbed and scatter in the patient's body.^9^ Scattered radiation levels near the patient can be relatively high, even under routine working conditions, and staff are subsequently exposed while conducting CV procedures.^1^, ^10^

There has been justifiable concern over the dose received by the physicians operating in this environment, but data detailing exposure to supporting staff during fluoroscopic procedures are scarce.^1^, ^11^, ^12^ The fundamental premise is to keep exposure to ionizing radiation as low as reasonably achievable (ALARA)^6^, ^13^ and organizations such as the International Commission on Radiological Protection (ICRP) recommend dose limits to those that are occupationally exposed.^14^ Staff radiation monitoring is performed as locally legislated to ensure that departments are complying with regulatory occupational dose limits, but problems with effective monitoring have been highlighted partly due to the attitude and radiation safety culture of staff.^15^ Poor adherence to the ICRP recommendation to conduct measurements using two dosimeters, one worn above and the other underneath the lead apron, as well as irregular use of personal dosimeters and has been emphasized,^16^ and it has been reported that appropriate dosimetry is essential to provide reasonable estimations of dose to the lens of the eye.^17^, ^18^, ^19^

There has been increasing concern over recent epidemiological evidence suggesting that radiation‐induced cataracts can occur at much lower doses than previously assumed.^20^, ^21^, ^22^ Staff involved in fluoroscopic CV procedures have demonstrated an elevated incidence of radiation‐associated lens changes.^16^, ^21^, ^23^, ^24^, ^25^, ^26^ In response, in 2011 the ICRP recommended reducing the occupational dose limit for the eye from 150 mSv (millisievert) to 20 mSv per year.^27^ This has resulted in numerous studies investigating the lens dose received by fluoroscopic operators, but there is very little research evaluating the risk of occupational eye exposure for nursing and allied health staff.^1^, ^11^, ^19^

Nurses are an integral part of the team conducting CV procedures, and many cases require staff to stand adjacent to the patient resulting in inadvertent exposure to radiation. To minimize the risk of exposure, it is vital that occupational dose to individuals is monitored and quantified. To date, the occupational exposure to nurses within the CV setting is widely unexplored.

### 1.A Review objective

The purpose of this review is to provide a current account of research specifically examining occupational dose to nursing staff during x‐ray guided CV procedures. It will compare results of publications within procedural contexts, critically review the findings, and assess areas in which further research would be beneficial.

## 2 MATERIALS AND METHODS

A search for relevant literature published between 2011 and 2017 was undertaken between November 2016 and June 2017 to retrieve articles related to occupational radiation dose to nursing staff present during fluoroscopically guided CV procedures. A combination of keywords was used correlated to occupational radiation dose to nurses, i.e.: “nurse occupational dose”, “nursing fluoroscopy”, “staff fluoroscopy dose”, and “occupational fluoroscopy dose”. Search terms were purposefully general to ensure that articles which did not explicitly articulate ‘cardiovascular’ terminology were included in the initial screening for suitability for inclusion in the review. Due to the relatively small number of identified studies, reference lists of located manuscripts were also used to detect additional articles. Due to the rapid advancements in both imaging and procedural equipment in the last decade, searches were limited to those published after 2010 to ensure relevance to current operating practices.

**Figure 1:**
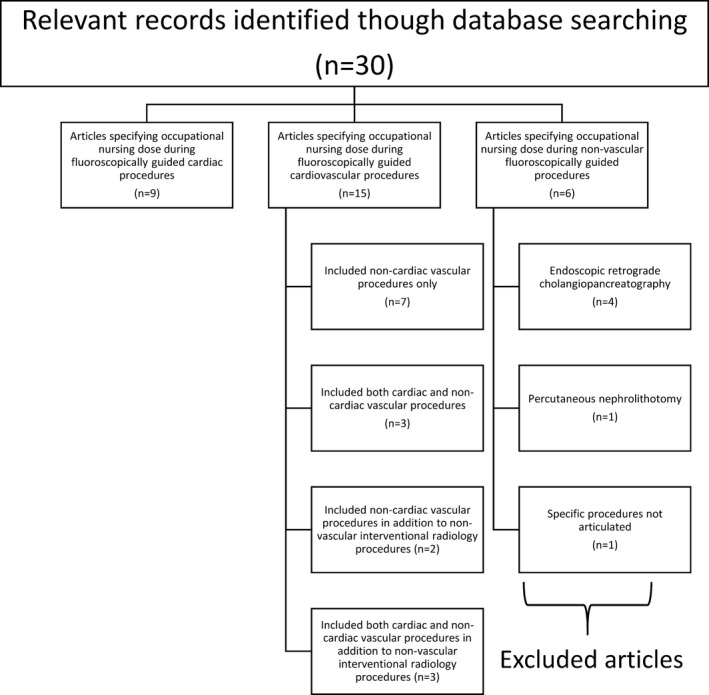
Flow diagram of study selection and exclusion process.

A total of thirty potentially relevant articles were identified and of these six articles were excluded from the review as the investigated radiation doses to nurses were not directly related to the imaging of the CV system as illustrated in Fig. 1. The literature was subsequently reviewed, analyzed, and compared. A summary of selected articles is provided in Table 1.

**Table 1:** Summary of reviewed literature

First author; year; location	Studied population	Cases	Collection period	Phantom measurements	Clinical	Intervention
Domienik, J. (2012) Poland^1^	Cardiologist* Nurse*	Vas IC (D + I) (n = 79) RFA (n = 11) PPM/ICD (n = 20) CRT/CRT‐D (n = 11)	*	y‐ for calibration of dosimeters Hp(0.07)	y	n
Chohan, M. (2015) United States of America^11^	Patient (n = 24) Radiologist (n = 1) Scout nurse*	Vas NR D (n = 18) I (n = 6)	July 2011 to Dec. 2011	n	y	n
Chida, K. (2013) Japan^12^	IR physician (n = 18) nurse (n = 7) Radiographer (n = 8)	Vas IC D (n = 5280) I (n = 1326)	During 2009	n	y	n
Antic, V (2012) Serbia^19^	Primary operator (n = 13) Secondary operator (n = 8) Scrub nurse (n = 18) Radiographer (n = 12)	Vas IC (D + I) (n = 106)	*	n	y	n
Sailer, A. ( 2015) *^25^	Primary operator* Second operator* Scrub nurse* Scout nurse* Radiographer* Anaesthesiologist*	EVAR (n = 22) TEVAR (n = 11) FEVAR (n = 11)	Sept. 2013–Jan. 2014	n	y	n
Nuraeni, N. (2016) Indonesia ^29^	Radiologist (n = 1) Scrub nurse (n = 1) Scout nurse (n = 1) Radiographer (n = 1)	Vas NR (D + I) (n = 8)	*	n	y	n
Mohapatra, A. (2013) * ^31^	Primary operator Secondary operator Total (n = 101) Scrub nurse * Radiographer *	FEVAR (n = 39)	Oct. 2011–Feb. 2012	n	y	n
Korir, G. (2012) Kenya ^32^	Physician* Nurse* Radiographer* Neurologists* Clinical staff* Total (n = 216)	Vas INR Vas IC (D + I) (n = 54)	Nov. 2007–end time *	n	y	n
Omar, A. (2017) Sweden ^34^	IR and IC physician (n varied per room) Scrub nurse Scout nurse Anaesthetist Anaesthetic nurse	Vas IR, IC and INR NVas IR R1 (n = 200) R2 (n = 55) R3 (n = 80) R4 (n = 10)	R1 (hybrid IR OR)—11 months R2 (IR)—2 months R3 (IC)—3 months R4 (INR)—3 months	n	y	n
Racadio, J (2014) ^35^	IR physician (n = 4) IR fellow (n = 4) Nurse ^ (n = 3) Radiographer (n = 7) Anaesthetist *	Vas IR (n = 38) NonVas IR (n = 207) CP (n = 97) OP (n = 148)	CP–12 weeks OP–17 weeks	n	y	CP–blinded OP–unblinded
Baumann, F. (2015) * ^36^	IR physician and fellows* Scout nurse ^ * Radiographer * Anaethetist *	Vas and NonVas IR (D + I) CP (n = 88) LP (n = 50) OP (n = 114)	CP—6 weeks LP—6 weeks OP—10 weeks year *	n	y	CP—blinded LP—unblinded, not evaluated OP—unblinded and evaluated
Sandblom, V. (2013) Sweden ^37^	Cardiologist (n = 3) Nurse (n = 10)	Vas IC (D + I) CP (n = 80) OP (n = 81)	CP—1 month OP—1 month	n	y	CP—blinded OP—unblinded
James, R. (2015) United States of America ^38^	Radiologist (n = 2) Scrub nurse* Scout nurse* Total (n = 25)	Vas NR (D) CP (n = 60) OP (n = 60)	Apr. 2012–Aug. 2013	n	y	CP—blinded OP—unblinded
Butcher, R. (2015) Australia ^39^	Scrub nurse* Scout nurse* Total (n = 10)	Vas IR (D + I) CP (n = 28) OP (n = 28)	*	n	y	CP—blinded OP—unblinded
Haga, Y. (2017) Japan ^44^	Cardiologist (n = 12) Nurse (n = 11)	Vas IC (D) (n = 1707) Vas IC (I) (n = 902)	Sept. 2015–Feb. 2016	n	y	n
Gilligan, P. (2015) * ^45^	Cardiologist (n = 14) Nurse ^ * Cardiac Technicians * Radiographer *	IC (total n*)	3 times within 7 months	n	y	P1—standard shield P2—larger shield with lamellae and femoral cutout + additional flexible shield
McLean, D. (2016) * ^46^	Cardiologist * IC nurse ^ * IR operator (n = 6) IR nurse ^ (n = 9) IR radiographer (n = 2) ERCP operator * ERCP nurse ^ *	Vas IR (n = 93) IC (n = 192) ERCP (n = 34)	1 month per location	n	y	n
Efstathopoloulos, E. (2011) Greece ^47^	Cardiologist (n = 5) Radiologist (n = 5) Nurse (n = 3)	IC (D) (n = 6) PPM (n = 1) Vas IR (D + I) (n = 11)	Oct. 2008—Jan. 2009	n	y	n
Omar, A. (2015) Sweden ^48^	Cardiologist (n = 1) Nurse (n = 3)	IC *	1 month	y	y	n
Rigatelli, G. (2016) Italy ^49^	Physician (n = 4) Nurse (n = 9) Radiographer (n = 7)	IC (D + I) (n = 2130) Vas peripheral (D + I) (n = 440) INR (n = 60)	12 months (2014)	y	y	n
Principi, S. (2015) Spain ^52^	P1—cardiologist (n = 9) P1—nurse ^ (n = 6) P2—cardiologist (n = 3) P2—nurse ^ (n = 1)	Vas IC (D + I) *	P1—2 weeks P2—7 weeks	n	y	n
Urboniene, A. (2015) Lithuania ^53^	IC physician (n = 114) IC nurse (n = 137)	Vas IC (n*) Non Vas IC (n*)	2012‐2013 1 month for the eyes	n	y	n
Komemushi, A. (2014 * ^63^	IR physician (n = 3) Nurse (n = 5) ED physician (n = 1)	Vas IR Non Vas IR CG (n = 50) NCG (n = 43)	Mar.—May 2012	n	y	CG—nurse alerted operator before approaching patient NCG—no alert
Mori, H. (2015) Japan ^64^	IR nurse (n = 27) IC nurse (n = 42)	Vas IR (n*) Vas IC (n*)	*	n	y	P1—change dosimeters P2—staff education P3—additional portable lead shields P4—reducing radiation parameters

Summary of review literature. RFA: radiofrequency ablation; PPM: permanent pacemaker; ICD: implantable cardioverter defibrillator; CRT: cardiac resynchronization therapy; EVAR: endovascular aortic repair; TEVAR: thoracic aortic repair; FEVAR: fenestrated aortic repair; INR: interventional neuroradiology; NR: neuroradiology; IC: interventional cardiology; Vas: vascular; D: diagnostic; I: interventional; CP: closed phase; OP: open phase; LP: learning phase; R: room; OR: operating room; P1: Phase 1; P2: Phase 2; P3: Phase 3; P4: Phase 4; ERCP: endoscopic retrograde cholangio‐pancreatography; CG: call group; NCG: no call group; ^: role not articulated; *: not articulated.

### 2.A Radiation dose monitoring

It has been demonstrated that the dose to nursing staff during fluoroscopic procedures can be similar or higher than that received by the physician^28^, ^29^, ^30^ with evidence of an increasing trend toward higher dose levels to nurses working in this environment.^28^ It is therefore important to quantify the radiation exposure to individuals working within fluoroscopic departments.^31^, ^32^, ^33^

**Figure 2:**
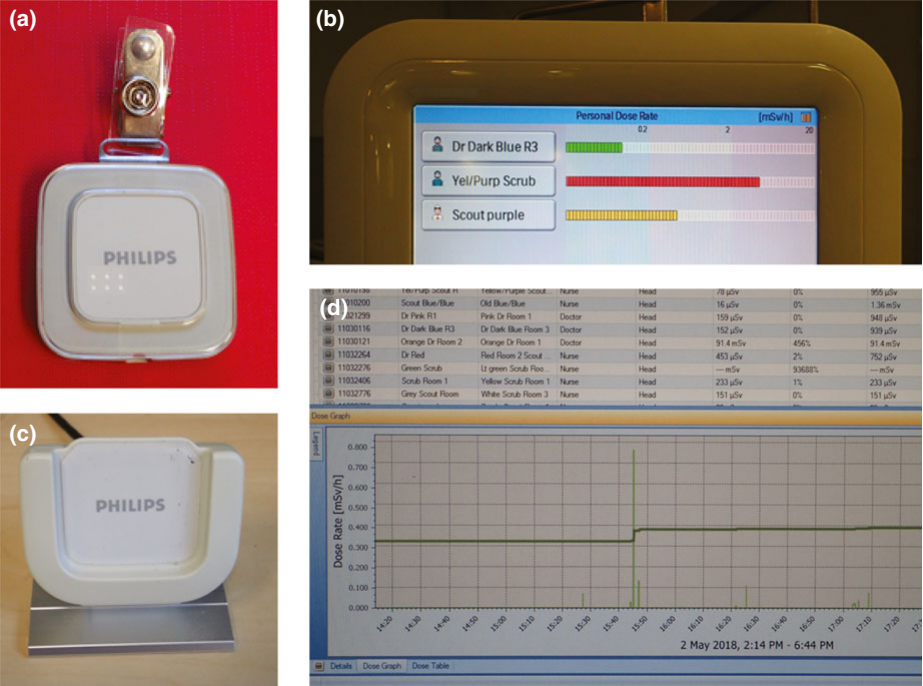
Components of a real time feedback monitoring system. (a) personal dosimeter. (b) base station. (c) download cradle. (d) dose manager software.

Typically, the devices used to evaluate the individual cumulative radiation exposure are personal dosimeters, which are usually badges worn by occupationally exposed staff during procedures. The ICRP recommends the proper use of personal monitoring badges in interventional fluoroscopic laboratories to monitor and audit occupational radiation dose.^14^ There was a variety of styles, anatomical positioning, and calibration of dosimeters utilized in the reviewed literature (Table 2). Active dosimetry systems, such as DoseAware (Philips Medical Systems, Amsterdam, The Netherlands) provide real time visualization of radiation dose rate. It consists of a personal dosimeter worn by staff [Fig. 2(a)], a wireless base station which displays live radiation exposure information transmitted from individual dosimeters [Fig. 2(b)], a download cradle [Fig. 2(c)], and computer software which downloads badge data for analysis [Fig. 2(d)]. Several studies evaluated the effectiveness of immediate exposure information on staff behavior by monitoring dose received by DoseAware^31^, ^34^, ^35^, ^36^, ^37^, ^38^ or other real time systems.^39^ The blinded, or closed phase measurements were downloaded from badges worn when staff were not able to view the base station display. During the unblinded, or open phase staff could visualize the real time dose rate information on the base station and modify behavior.

**Table 2:** Location, calibration, and dose values of dosimeters

Reference: First author (year)	Cases	Monitored staff‐1	Badge location	Calibration	Dosimeter type	Monitored staff‐2	Badge location	Calibration	Dosimeter type	Monitored staff‐3	Badge location	Calibration	Dosimeter type	Monitored staff dose‐1	Monitored staff dose‐2	Monitored staff dose‐3
Domienik, J. (2012) ^1^	Vas IC Non Vas IC	Nurse (n*)	1—FH (E) 2—RF L 3—RF R 4—W L 5—W R 6—K L 7—K R 8—An L 9—An R	Hp(0.07)	TLD	Cardiologist *	1—FH (E) 2—RF L 3—RF R 4—W L 5—W R 6—K L 7—K R 8—An L 9—An R	Hp(0.07)	TLD					MD/case# 1—15.7μSv 2—26 μSv 3—24.3 μSv 4—24.6 μSv 5—23.7 μSv 6—14.5 μSv 7—13 μSv 8 & 9—33.3 μSv	MD/case# 1—67.6 μSv 2—203 μSv 3—205 μSv 4—133 μSv 5—115 μSv 6—72.8 μSv 7—43.1 μSv 8 & 9 –108 μSv	
Chohan, M. (2015) ^11^	Vas INR	Scout nurse (n*)	1—eye R (E)	*	ED	Radiologist (n = 1)	1—eye L (E) 2—eye L (U)	*	ED	pt	1‐head (E)	*	TLD	1‐MD/case 30 + /‐ 60 μSv	1—MD/case 80 + /‐ 190 μSv 2 –MD/case 5 + /‐ 16 μSv	cranial entrance MD/case 220270 + /‐221170 μSv
Chida, K. (2013) ^12^	Vas IC	Nurse ^ (n = 7)	1—Ch (U) 2—Co (E)	Hp(10)	PGD	Physician (n = 18)	1—Ch (U) 2—Co (E)	Hp(10)	PGD	rad (n = 8)	1—Ch (U) 2—Co (E)	Hp(10)	PGD	annual MD equiv./year 4730 + /‐720 μSv	annual MD equiv./year 19840+/‐12450 μSv	annual MD equiv./year 1300 + /‐ 1000 μSv
Antic, V (2012) ^19^	Vas IC	Scrub nurse (n = 18) second physician (n = 8)	1—eye L (E)	Hp(3)	APD	Cardiologist (n = 8)	1—eye L (E)	Hp(3)	APD	rad (n = 12)	1—eye L (E)	Hp(3)	APD	MD/case 1—33 μSv	MD/case 1—121 μSv	MD /case 1—12μSv
Sailer, A. ( 2015) ^25^	EVARS (angio)	Scrub nurse (n*)	1‐Ch (E)	Hp(10)	APD	Radiologist (n*) Cardiologist (n*) Gastroenterologist (n*)	1—Co (E)	Hp(10)	APD	Scout nurse (n*)	1—Co (E)	Hp(10)	APD	MD/case 17 μSv	MD/case 170 μSv	MD/case 4 μSv
Nuraeni, N. (2016) ^29^	Vas INR	Nurse (n = 2) A & B	1—eye side * (E)‐A only 2—Ch (U) 3—Co (E) 4—Co (U) 5—G (E) 6—G (U) 7—F (E)‐A only	1‐ Hp(3) 2 to 6‐Hp(10) 7‐ Hp(0.07)	TLD	Radiologist (n = 1)	1—eye side * 2—Ch (U) 3—Co (E) 4—Co (U) 5—G (E) 6—G (U) 7—F (E)	1—Hp(3) 2‐6 Hp(10) 7—Hp(0.07)	TLD	rad (n = 1)	1—eye side * 2—Ch (U) 3—Co (E) 4—Co (U) 5—G (E) 6—G (U) 7—F (E)	1‐ Hp(3) 2 to 6‐Hp(10) 7—Hp(0.07)	TLD	Highest dose 2—2 μSv (A)	Highest dose 2—1 μSv	Highest dose 2—1 μSv
Mohapatra, A. (2013) ^31^	EVARS	Scrub nurse (n*)	1‐Co (E)	Hp(10)	APD	Primary & Assistant surgeons (n = 101)	1—Co (E)	Hp(10)	APD	Dosimeter on equipment	1‐on anaethetic equipment	Hp(10)	APD	MD/case 26μSv	MD/case 125μSv	MD/case 268μSv
Korir, G. (2012) ^32^	Vas IC Vas IR	Nurse ^ (n*)	1—Co (E)	*	TLD	Cardiologist (n*) Radiologist (n*)	1—Co (E)	*	TLD	rad (n*)	1—Co (E)	*	TLD	MD/case‐ 270 μSv	MD/case‐ 340 μSv	MD/case—220 μSv
Omar, A. (2017) ^34^	Vas IC Non Vas IC R1 & 2 Vas IC R3 Vas INR R4	Scrub nurse (n*)	1—Ch (E)	Hp(10)	APD	Radiologist (n > 14) Cardiologist (n = 6)	1—Ch (E)	Hp(10)	APD	Scout nurse (n*)	1—Ch (E)	Hp(10)	APD	equiv. eye dose/case calculated from T APD# R1‐13 μSv R2‐51 μSv R3—5.7 μSv R4‐11 μSv	equiv. eye dose/case calculated from T APD# R1—60 μSv R2—190 μSv R3—66 μSv R4—8.6 μSv	equiv. eye dose/case calculated from T APD# R1—2.7 μSv R2—8.9 μSv R3—5.7 μSv R4—3.0 μSv
Racadio, J (2014) ^35^	Vas IR Non Vas IR	Nurse ^ (n = 3)	1—Ch (E)	Hp(10)	APD	Radiologist (n = 4) N or F from pt	1—Ch (E)	Hp(10)	APD	IR fellow (n = 4)	1—Ch (E)	Hp(10)	APD	*	CP median~ N—0.15μSv/min F—4.14μSv/min OP median~ N—0.02μSv/min F‐4.12μSv/min	CP median~—0.0μSv/min OP median~—0.0μSv/min
Baumann, F. (2015) ^36^	Vas IR Non Vas IR	Nurse ^ (n*)	1—Co (E)	Hp(10)	APD	Radiologist (n*)	1—Co (E)	Hp(10)	APD	Anaethetist (n*)	1—Co (E)	Hp(10)	APD	Avg. of all staff (drs, nurse, primary physician, fellow, rad)~ CP—42.79 μSv/min OP—19.81 μSv/min		anaethetist*~ CP—16.9 μSv/min OP—8.9 μSv/min
Sandblom, V. (2013) ^37^	Vas IC	Scrub nurse (n = 10) CP‐69 cases OP–73 cases	1—Ch (E)	Hp(10)	APD	Cardiologist (n = 3)	1—Ch (E)	Hp(10)	APD					median/case CP—4.3 μSv OP—2.5 μSv	median/case CP—9.9 μSv OP—8.5 μSv	
James, R. (2015) ^38^	Vas INR	scrub nurse (n=<26) CP—60 cases OP—60 cases	1—Ch (E) 2—Ch (E)	1—* 2‐Hp(10)	1—TLD 2—APD	Radiologist (n = 2) A & B OP—30 cases each CP—30 cases each	1—Ch (E) 2—Ch (E)	1—* 2‐Hp(10)	1—TLD 2—APD	Scrub nurse (n=<26) CP—60 cases OP—60 cases	1—TLD 2—APD	1—* 2—Hp(10)	1—TLD 2—APD	CP MD‐0.045 μSv/Gy‐cm2 OP MD‐0.02 μSv/Gy‐cm2 dose divided by DAP	A‐CP MD ‐0.028 μSv/Gy‐cm2 A‐OP MD‐0.051 μSv/Gy‐cm2 B‐CP MD ‐0.243 μSv/Gy‐cm2 B‐OP MD‐0.069 μSv/Gy‐cm2 dose divided by DAP	B‐CP MD‐0.033 μSv/Gy‐cm2 B‐OP MD‐0.015 μSv/Gy‐cm2 dose divided by DAP
Butcher, R. (2015) ^39^	Vas IR	Scrub nurse (n = 10) CP—14 cases OP—14 cases	1—Ch (U)	*	SM					Scout nurse (n = 10) CP—12 cases OP—12 cases	Ch (U)	*	SM	MD/case CP—2.18 μSv OP—0.674 μSv		MD/case CP—3.25 μSv OP—0.009 μSv
Haga, Y. (2017) ^44^	Vas IC	Nurse ^ (n = 11)	1—eye L (E) 2—Co (E)	1—Hp(3) 2—Hp(0.07)	1—TLD 2—PGD	Cardiologist A—with LG (n = 9) B—without LG (n = 3)	1—eye L (E) 2—eye L (U) A only 3—Co (E)	1—Hp(3) 2—Hp(3) 3—Hp(0.07)	1—TLD 2—TLD 3—PGD					est. annual dose 1—3300 + /‐2000 μSv 2—4000 + /‐2400 μSv	est. annual dose A 1 –15800 + /‐ 6600 μSv 2—6200 + /‐ 2600 μSv 3—22800 + /‐ 12800 μSv est. annual dose B 1—12600 + /‐ 10200 μSv 3—10000 + /‐5200 μSv	
Gilligan, P. (2015) ^45^	Vas IC Non Vas IC	Scrub nurse (n*) P1—Standard shield P2—larger shield + pt drape	1—Co (E)	Hp(10)	EPD	Cardiologist (n = 14) P1—standard shield P2—larger shield + pt drape	1‐Co (E)	Hp(10)	EPD	rad (n*) P1—standard shield P2—larger shield + pt drape	1‐ Co (E)	Hp(10)	EPD	median P1—1 μSv P2—0.1 μSv	median P1—15.4 μSv P2—7.3 μSv	median P1—4.2 μSv P2—2.5 μSv
McLean, D. (2016) ^46^	Vas IR IC ERCP	Nurse A—Cardiology (n*) B—Angiography (n = 9) C—ERCP cases (n*)	1—eye L (E)	Hp(3)	TLD	A—cardiologist (n*) B—radiologist (n = 6) C—gastroenterologist (n*)	1—eye L (E)	Hp(3)	TLD					median eye dose! A‐130 μSv B‐100 μSv C‐360 μSv	median eye dose! A‐340 μSv B‐930 μSv C‐1510 μSv	
Efstathopoloulos, E. (2011) ^47^	IC PPM Vas IR	Nurse ^ (n = 3)	1—eye L (E) 2—central FH (E) 3—MF L (E) 4—W L (E) 5—MF R (E) 6—W R (E) 7—leg L (E) 8—leg R (E)	H_p_(0.07)	TLD	Radiologist (n = 5) Cardiologist (n = 5)	1—eye L (E) 2—central FH (E) 3—MF L hand (E) 4—L W (E) 5—MF R hand (E) 6—R W (E) 7—L leg (E) 8—R leg (E)	H_p_(0.07)	TLD					MD/case 1—1μSv 2—4μSv 3—4μSv 4—26μSv 5—2μSv 6—26μSv 7—15μSv 8‐18μSv	MD/case 1—37μSv 2—64μSv 3—324μSv 4—485μSv 5—88μSv 6—108μSv 7—124μSv 8‐103μSv	
Omar, A. (2015) ^48^	Vas IC	Nurse ^ (n = 3)	1—eye R & L (E) 2—Ch (E) 3—central FH (E)	Hp(10)	1—TLD 2—APD 3‐APD	Cardiologist (n = 1)	1—eye R & L (E) 2—Ch (E) 3—central FH (E)	Hp(10)	1—TLD 2—APD 3‐APD					quantitative measurements *		
Rigatelli, G. (2016) ^49^	IC Vas IR INR	A‐nurse <165 cm tall (n = 6) B‐nurse >165 cm tall (n = 3)	1‐Ch (E)	*	TLD	A‐physician <165 cm tall (n = 2) B‐ physician >165 cm tall (n = 2)	1—Ch (E)	*	TLD	A‐rad <165 cm tall (n = 4) B‐rad >165 cm tall (n = 3)	1—Ch (E)	*	TLD	Annual mean for all staff A—4550 + /‐ 4000 μSv B—1950 + /‐ 1000 μSv		
Principi, S. (2015) ^52^	Vas IC	Nurse ^ P1 (n = 6) P2 (n = 1)	1—eye L (E) 2—Ch (E)	1—Hp(3) 2‐Hp(10)/Hp(0.07)	TLD	Cardiologist P1 (n = 9) P2 A (n = 2) B (n = 1)	1—eye L (E) 2—Ch (E) P2B—eye L (E & U)	1—Hp(3) 2‐Hp(10)/Hp(0.07)	TLD					nurse MD/case# P1‐17 μSv P2—13 + /‐5 μSv	dr MD/case# P1‐ 114 μSv P2—97 μSv P2B—mean U/E—3.5	
Urboniene, A. (2015) ^53^	Vas IC Non Vas IC	Nurse ^ 1 & 2 (n = 137) 3 (n = 8)	1—T (U) 2—Co (E) 3—eye (E)	1—Hp(10) 2—Hp(10) 3—Hp(3)	TLD	Physician 1 & 2 (n = 114) 3 (n = 42)	1—T (U) 2—Co (E) 3—eye (E)	1—Hp(10) 2—Hp(10) 3—Hp(3)	TLD					Avg. annual dose# 2 –1490 μSv (Avg. over 9 hospitals) est. eye dose/year# 3 ‐1 600 μSv/yr	Avg. annual dose# 2—14500 μSv (Avg. over 9 hospitals) est. eye dose/ year# 3‐ 2300 μSv/yr (Avg. over 18 physicians)	
Komemushi, A. (20a14)^63^	Vas IR Non Vas IR	Scout nurse (n = 5) A—No CG B—CG	1—Ch (E) 2—T (U)	Hp(10)	PDM	Radiologist (n = 4) A—No CG B—CG	1—Ch (E) 2—T (U)	Hp(10)	PDM					MD/case 1A—0.51 + /‐ 1.17 μSv (A) 1B ‐0.16 + /‐ 0.41 μSv (B) 2A & B—below detectable limit (A & B)	MD/case 1A—8.70 + /‐ 12.70 μSv 1B ‐8.88 + /‐ 13.38 μSv 2A—0.65 + /‐ 1.45 μSv 2B‐0.48 + /‐ 1.03 μSv	
Mori, H. (2015)^64^	Vas IC Vas IR	Vascular IR nurse (n = 69)	1—T (U) 2—Co (E)	Hp(10)	PGD									a reduction of annual effective dose to approx 1/3 or baseline dose after education, and a reduction to 2/5 of baseline after reduction in pulse rates		

Vas: vascular; Non Vas – non vascular radiology procedures; IC: interventional cardiology; IR: interventional radiology; INR: interventional neuro‐radiology; EVAR: endovascular aortic repair; Ch: chest; Co: collar; T: trunk; W: wrist; K: knee; F: finger; RF: ring finger; MF: middle finger; G: gonad; FH: forehead; An: ankle; L: left; R: right; P: phase; (E): external to protective equipment; (U): under protective equipment; APD: active personal dosimeter; ED: electronic dosimeter; EPD: electronic personal dosimeter; PDM: personal dosimeter; PGD: phosphate glass dose meter; SM: survey meter; TLD: thermoluminescent dosimeter; CP: closed phase; OP: open phase; LP: learning phase; LG: lead glasses; CG: call group; NCG: no call group; rad: radiographer; pt: patient; dr; doctor; Avg.: average; MD: mean dose; DAP : dose area product; μSv:microsievert; Gy.cm2 : gray‐centimetres squared; N: near; F: far; *: not articulated; ^: role not articulated; #: average calculated from data; ~: normalized with fluoroscopy time; !: dose normalized by cumulative KAP; equiv.: equivalent; est.: estimated.

Baumann et al. report the overall mean staff dose per fluoroscopic minute was 42.79 vs 19.81 μSv/min (*P* < 0.05) comparing the closed and open phases,^36^ and Racadio et al. also demonstrate that the dose to staff was higher in the closed phase with a median of 3.01 μSv/min than in the open phase 0.56 μSv/min.^35^ Similarly, Butcher et al. reports a mean personal percentage dose reduction for scrub nurses from 0.065% (SD, 0.12) in the closed phase to 0.03% (SD, 0.034) in the open phase, while scout nurses decreased from 0.06% (SD, 0.11) measured during the closed phase, to 0.009% (SD, 0.01).^39^ None of these reductions were reported as statistically significant with one cited explanation the possibility that the nurses had a restricted view of the readout monitor during cases, but it is acknowledged that real time dose feedback can be effective in dose reduction.^35^, ^36^, ^37^, ^38^, ^39^

### 2.B The effect of equipment and staff location

**Figure 3:**
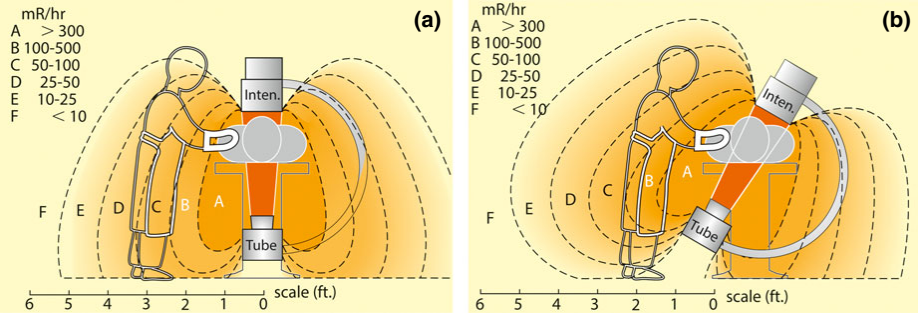
Exposure levels during fluoroscopy. (a): straight under table tube orientation. (b): central ray 30° from vertical. Reprinted with permission from Personnel exposure during fluoroscopy procedures, Postgraduate Radiology 8:162–173, 1988. 1 millirem (mR) is equivalent to 0.01 millisievert (mSv).

Radiation scatter is the primary mechanism of operator and staff exposure, and understanding the factors that can affect its magnitude and distribution is essential.^40^ As X‐ray scatter from the patient is the primary source of radiation dose to in‐room personnel,^41^ staff location within the fluoroscopy room influences the level of occupational exposure.^1^, ^19^, ^42^ In x‐ray guided CV procedures, the area of greatest scatter alters as the geometry of the x‐ray tube changes (Fig. 3).^43^ Nursing staff may undertake several roles within fluoroscopic suites, and the in‐room location of the nurse may vary during procedures. In many of the reviewed articles, the role of the nurse was not well‐defined and it was unclear whether staff were performing the scrub or scout role^12^, ^32^, ^35^, ^44^, ^45^, ^46^ and consequently reported data may represent an average of the dose of both duties.

Mohapatra et al. investigated several staff roles and found that there was surprising variation in doses to different personnel present during the same procedure.^31^ The authors also identified that personal behavior within the fluoroscopic suite alters dose considerably. Depending on their responsibilities during the procedure nurses may have greater opportunity of deliberately increasing their distance from the patient resulting in a decrease in dose.^1^, ^25^, ^29^, ^39^

Some authors investigated dose in relation to proximity to the x‐ray tube.^25^, ^34^, ^38^, ^47^, ^48^, ^49^ Explanatory diagrammatic representation of the position of staff was provided in several articles^25^, ^38^, ^47^, ^48^, ^49^ which allows comparison by dosimetric location rather than assigned role. Specific articulation of staff distances from the x‐ray tube or table^31^, ^47^, ^49^ was constructive when comparing occupational doses.

### 2.C Lead shielding

Lead shielding refers to the use of lead, or lead equivalent products to shield staff from radiation. Variations in accessibility and utilization of lead shielding devices by staff in fluoroscopic suites have been well documented^50^, ^51^ and this has been reflected in reported use of personal protection in the reviewed studies (Table 3). Thyroid shields were either not worn^12^, ^44^ or inconsistently worn by staff at some centers.^52^ Only one reviewed article specifically articulated the use of a lead skull cap during fluoroscopic procedures and was utilized by the operator only.^11^ Lead glasses also had varying degrees of use with several studies reporting that while doctors routinely used lead eye protection, nursing staff did not.^11^, ^19^, ^44^, ^47^, ^53^

**Table 3:** Protective equipment utilized by staff

Reference: First author (year)	Lead coat	Thyroid shield	Lead glasses	Table mounted lead drape	Ceiling mounted lead shield	Lead cap	Lead gloves	Additional shielding	X‐ray tube orientation
Domienik, J. (2012) ^1^	y	*	*—nurses y—drs (use varied)	R1—y R2—y R3—use varied R4—use varied R5—n R6—y	R1—y R2—use varied R3—use varied R4—n R5—n R6—n	n	*	*	OT (R5) UT (R1‐4;6)
Chohan, M. (2015) ^11^	y	y	y	y	y	n‐ nurses y—drs	n	Additional lead shield on anaesthetic side	Biplane
Chida, K. (2013) ^12^	y	n	*	y	y	n	*	*	UT
Antic, V (2012) ^19^	y	y	0%—nurses 46%—doctors	y	y	n	n	nil	UT (x2)
Sailer, A. ( 2015) ^25^	y	y	n	n	n	n	n	nil	UT
Nuraeni, N. (2016) ^29^	y	y	*	y	y	*	*	*	biplane
Mohapatra, A. (2013) ^31^	y	y	y—use varied	y	y (2)	n	*	Floor shield for anaethetic team (infrequently used)	UT
Korir, G. (2012) ^32^	*	*	*	*	*	n	*	*	*
Omar, A. (2017) ^34^	y	*	*	R1—y (x2) R2—y R3—y R4—y	R1 (hybrid IR OR)—y (x2) R2 (IR)—y (x2) R3 (IC)—y (large) R4 (IR)—y	*	*	Mobile full body radiation protection shield available in R1, 3 and 4	UT (R1‐3) biplane (R4)
Racadio, J (2014) ^35^	y	*	*	y—use varied	y—use varied	*	*	*	UT
Baumann, F. (2015) ^36^	y	y	n	*	*	n	n	*	*
Sandblom, V. (2013) ^37^	y	*	*	*	y	*	*	*	*
James, R. (2015) ^38^	*	*	*	*	y	*	*	Standing stationary full body length leaded acrylic barrier	Biplane
Butcher, R. (2015) ^39^	y	*	*	*	*	*	*	*	UT
Haga, Y. (2017) ^44^	y	n	0%—nurses 75%—drs	*	n	n	n	Nil	UT
Gilligan, P. (2015) ^45^	*	*	n—nurses y—drs (use varied)	*	y	n	*	*	UT
McLean, D. (2016) ^46^	*	*	50%—IC staff 30%—Vas IR staff 0%—ERCP staff	*	y—IC y—IR Angiography n—ERCP	n	n	*	UT
Efstathopoloulos, E. (2011) ^47^	y	y	0%—nurses 71%—radiologists 83%—cardiologists	y	y	n	Available but not used	Mobile floor screen 78%—IR 14%—IC	UT
Omar, A. (2015) ^48^	y	*	y!	*	y	y	*	*	UT
Rigatelli, G. (2016) ^49^	y	*	*	*	Phantom measurements taken with and without CML	*	*	*	UT
Principi, S. (2015) ^52^	y	17%—nurses 100%—drs	0%—nurses 11%—drs	y	y—78%	n	n	nil	UT
Urboniene, A. (2015) ^53^	y	y	50%—IR staff	*	y—use varied ~ 76% of workers were protected with lead screens or glasses	n	*	*	*
Komemushi, A. (2014)^63^	y	*	*	y	y	n	*	*	UT
Mori, H. (2015) ^64^	y	*	*	y	*	*	*	Portable radiation shielding screens	UT

Vas: vascular; R: room; CML: Ceiling mounted lead; IC: interventional cardiology; IR: interventional radiology; ERCP: endoscopic retrograde cholangiopancreatography; drs: doctors: UT: undertable; OT: overtable; OR: operating room; !: ?protective glasses’ unclear whether this is lead or plastic; * : not articulated; y: yes; n: no.

**Figure 4:**
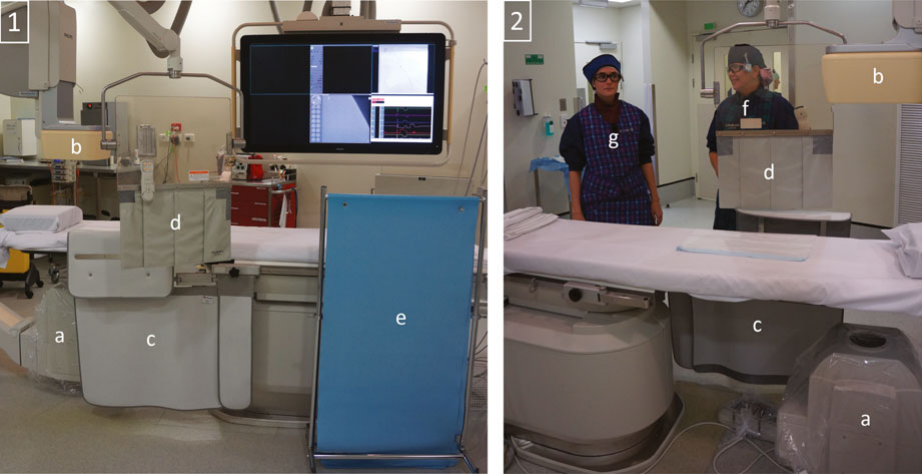
Lead protection and staff position: 1:View from operating side; 2: View from non‐operating side; (a) x‐ray tube; (b) x‐ray detector; (c) Table mounted lead drapes with extension panel; (d) Movable ceiling mounted lead glass shield with lead drapes; (e) Moveable stand‐alone shield; (f) Common location for flurosocopic operator; (g) Common location for scrub nurse.

Consideration should also be given to the location of lead protection. This may include items such as ceiling mounted lead glass, table mounted, or stand‐alone lead shields (Fig. 4). This equipment provides a barrier between the scattered radiation from the patient and the staff member, but correct positioning is vital for effective dose minimization.^54^

The importance of careful positioning of the movable ceiling mounted lead shield has been previously reported^55^ especially when using biplane equipment,^56^ and this was echoed in the reviewed literature.^1^, ^11^, ^19^, ^25^, ^31^, ^32^, ^34^, ^35^, ^46^, ^48^, ^52^, ^53^ Several authors declared the absence of ceiling^25^, ^44^, ^46^ and table‐mounted lead shields^25^ when no other additional lead protection such as lead glasses or skull caps were worn by staff.^25^, ^44^ It has been highlighted previously that some fluoroscopic staff have access to a ceiling mounted lead shield but choose not to utilize it^50^ and this was also found to be the case in a number of reviewed manuscripts.^1^, ^34^, ^35^, ^52^, ^53^

### 2.D Eye dose

While many dosimeters are worn underneath protective lead aprons, it is important to monitor dose for the unprotected areas of the body exposed to radiation.^19^ Ideally a dedicated dosimeter should be worn adjacent to the eye closest to the x‐ray tube and monitor lens dose using the operational quantity personal dose equivalent H_p_(3)^18^, ^56^, ^57^ which means it is designed to detect dose to the lens at a depth of 3 mm. Dosimeters are also available in H_p_(10) and H_p_(0.07) which estimate values for dose of deep organs and skin dose, respectively. Several of the reviewed manuscripts recorded eye dose at the level of the eye^1^, ^11^, ^19^, ^29^, ^44^, ^46^, ^47^, ^48^, ^53^, ^58^ and some utilized multiple dosimeters around the face or head (Table 2).^1^, ^11^, ^47^, ^48^, ^52^

Several studies positioned dosimeters external to protective lenses^19^, ^44^, ^46^, ^47^, ^48^ which gives an approximation of the unprotected dose to the eyes, but not the actual dose incident on the lens of the monitored staff member.^19^, ^46^, ^48^ To assess the benefit of protective lead glasses Haga et al. measured doses both inside and outside the lead eye ware and found the shielding effect was approximately 60% reduction in measured radiation dose in a clinical IC setting.^44^

Several of the reviewed research investigated whether eye dose to personnel would exceed the recommended ICRP dose limits. A number of authors found that staff eye doses were within acceptable limits, but it is observed that some of these findings relate to the pre‐2012 ICRP recommended limit of 150 mSv per year, not the revised limit of 20 mSv per year. With the new eye limits applied, nurses in Korir et al. study, with a mean dose of 270 μSv per case, and physicians in Domienik et al. and Efstathopoulos et al., with procedural eye doses of 67.6 and 64* *μSv, respectively, may be at risk of exceeding the current recommendations. Domienik et al. goes on to report an annual estimated eye dose for one operator of 247 mSv, which not only exceeds the new limit of 20 mSv, but definitively exceeded the old limit of 150 mSv. Mulitple reviewed studies highlighted the fact that this new eye dose limit could be exceeded by the operator when bad practices are followed, radiation protection tools are not used appropriately,^34^, ^47^ or when protective eyeglasses are not worn.^11^, ^19^, ^34^, ^44^, ^46^, ^52^, ^53^, ^59^, ^60^

With a recommended equivalent dose limit of 500 mSv in a year for the hands and feet, even the highest recorded average extremity dose of 485 μSv at the left wrist of a physician^47^ would require participation in over 1000 fluoroscopic cases within a year to be at risk of exceeding the recommended limit.

Chohan et al. demonstrated that scout nurses would receive 39 mSv of cumulative exposure per year and were at risk of exceeding the recommended ICRP eye limit^11^ and Antic et al. noted that a scrub nurse could exceed the limit if over 600 procedures per year were performed in this role.^19^ McLean et al.^46^ identified that the nursing staff received three of the highest six doses in the angiographic suite and noted that, while not routinely the closest to the patient, nurses were present during a large number of procedures. Chida et al. established that individual nurses were present for over double the number of coronary cases as interventionalists (average 754 ± 352 times vs 293 ± 145 times, respectively).^12^ Nuraeni et al. reported that a single monitored nurse, due to her proximity to the x‐ray tube and her habit of bowing her head during procedures, resulted in a similar eye exposure as the operator.^29^ If findings of nursing dose measured of 0.27 mSv per case at the collar in Korir et al.^32^ study were extrapolated, nurses would exceed the eye dose after only 75 cases.

### 2.E Imaging parameters

Mohapatra et al. found that digital subtraction angiography (DSA) acquisition runs, as opposed to fluoroscopy accounted for “a large fraction of individuals’ doses”^31^ (p. 702) which has been highlighted by other researchers.^61^, ^62^ James et al. reported changes in behavior regarding the use of DSA in cerebral angiography as a result of real time feedback from the scrub nurse's dosimeter which monitored a difference in the mean dose of 0.045 μSv/Gy‐cm^2^ during the closed phase, to 0.02 μSv/Gy‐cm^2^ during the open phase.^38^

It was demonstrated that reducing staff proximity to the x‐ray tube during fluoroscopic activation can be achieved by better communication between the operator and the nurse,^38^, ^63^ limiting DSA acquisitions^31^ and increasing staff distance during acquisitions especially when using large tube angles.^31^, ^38^ Adequate staff training and education were also seen as essential, and this was successfully supplemented by using real time feedback monitors.^34^, ^37^

### 2.F Staff education

Mori investigated nursing doses before and after staff were provided with practical education.^64^ This resulted in a decrease in annual effective dose from 1.33 to 0.47 mSv, which corresponds to similar studies.^65^, ^66^ Several authors articulated the need for appropriate training to heighten staff awareness to ideally result in the active participation of staff in optimizing occupational exposure.^32^, ^34^, ^35^, ^48^, ^52^, ^67^

## 3 DISCUSSION

While lead aprons were universally worn, it was concerning to note the irregular use of other radiation protection (Table 3). The use of lead glasses is especially important in the absence of a ceiling mounted lead shield and provides protection from the formation of radiation‐induced subcapsular cataracts.^33^ Although the reviewed literature was unconvincing in demonstrating a staff commitment to utilizing eye protection, a vast number of authors acknowledged the advantage of lead glasses,^1^, ^11^, ^19^, ^32^, ^34^, ^35^, ^44^, ^46^, ^48^, ^53^ and hopefully, this signals a trend toward greater compliance. Haga et al. report the mean ± the standard deviation for dosimeter measurements external to, and inside of protective lead glasses as being 7.9 ± 3.3 mSv and 3.1 ± 1.3 mSv/6 months, respectively, concluding the shielding effect was approximately 60%.^44^ The reviewed publications almost universally recommend the diligent use of appropriately positioned lead shielding and protective eyewear during fluoroscopic procedures.

Due to cardiac motion, DSA is infrequently used in cardiology procedures which may result in lower occupational doses as demonstrated by McLean et al.^46^ in reported lower extrapolated annual eye dose to nurses involved in fluoroscopic cardiac procedures (1.32 mGy) compared to vascular interventions (6.06 mGy). Authors investigating endovascular aortic repairs which, in theory, should expose staff to increased levels of radiation due to the proximity of staff to the irradiated area, the thickness of the imaged body part, and the use of DSA report mean nursing doses of 17 μSv (measured at the chest)^25^ and 26* *μSv (measured at collar level).^31^ Omar et al. (2017) report a higher equivalent eye dose received by nurses assisting during interventional neuroradiology procedures compared with the physician (11 vs 8.6 μSv).^34^

Ideally DSA runs should be limited where possible,^5^, ^31^, ^35^, ^36^, ^68^ magnification should be increased,^31^ and the pressure injector should be utilized to allow staff to stand further away from the patient during acquisitions.^31^, ^38^ James et al.^38^ reported modification of staff behavior during cerebral DSA due to real time monitoring. One physician substituted fluoro‐save where possible for visualization of the femoral artery, which has been shown to reduce dose by 95%.^62^ The pressure injector was more consistently used, as opposed to injecting by hand, thus allowing personnel to step back during DSA acquisitions which may have contributed to the significant decrease in mean dose for physician B from 0.243 μSv/Gy‐cm^2^ during the closed phase, to 0.069 μSv/Gy‐cm^2^ during the open phase. It was also reported that during the open phase the scrub nurses utilized the operating physician as a personal shield by stepping behind them to reduce exposure.^38^ Physicians should also let other in‐room staff know of an impending DSA acquisition so that the staff know to not approach the patient and stay behind shielding if possible.^38^, ^63^

Research indicates a considerable number of parameters which can cause a significant variation in resultant dose levels during fluoroscopic cases, even within the same type of procedures.^1^ The Optimization of RAdiation protection for MEDical (ORAMED) staff study also revealing a large variability of practices between cases and workplaces.^56^ Given the variation in procedure type, operator, tube geometry, and staff position, correlation of dose conditions within differing procedures proved difficult. This was exacerbated by the different reporting values used by the authors.

The ICRP notes that radiation training may be lacking which may result in a radiation safety issue for staff as well as patients^69^ and recommends that departments implement an effective optimization program through training and raising consciousness of radiology protection in individuals.^70^ The effectiveness in dose reduction to staff following radiation education has been highlighted^65^, ^66^, ^71^ as has the need for radiation training of occupationally exposed nursing staff.^72^

Several authors noted that nursing staff are at risk of exceeding recommended dose levels if radiation protection tools are not properly used. Given the variables that exist for nursing staff during fluoroscopic procedures, dose minimization is not as simple as increasing distance from the source of the scattered radiation. Given the invisible nature of radiation, staff should be provided with appropriate information and training to highlight factors which influence dose allowing them to become conscious contributors to personal dose minimization.

### 3.A Limitations of current evidence

Several limitations have been identified in the current literature. Many of the articles reviewed had relatively small sample sizes either due to the number of staff or procedures, or a relatively short data collection period. Evaluation of occupational nursing dose during fluoroscopic procedures is vital, and it is recommended that monitoring of nurse doses should be implemented as part of a robust quality assurance program. This review has highlighted the need for additional research to evaluate radiation exposure to nurses during fluoroscopic procedures. It would be constructive for future investigations to specifically articulate the location of the nurse during procedures and divide the monitoring per position as well as monitoring the dose to the individual. Having multiple dosimeters evaluating eye and extremity dose would also be beneficial.

### 3.B Strengths and limitations of the review

To the author's knowledge, this is the first review to examine literature reporting dose to nursing staff during fluoroscopic CV procedures. One limitation of the review is the difficulty in making direct comparisons of nursing dose in the reviewed studies due to the variability of staff role and position, the wide variety of procedures, the type, calibration, and location of the dosimeters and the differing parameters in the reporting of dose.

## 4 CONCLUSION

This literature review was undertaken to highlight research specifically investigating the occupational dose received by nursing staff within fluoroscopic examinations and to critically review the findings. Nursing staff should be aware of the effect that x‐ray tube angle, orientation, and acquisition type has on potential exposure and use this knowledge to position themselves and lead shielding correctly to minimize risk. Appropriate education and training should be provided to inform nursing staff working within CV fluoroscopic suites of dose reduction techniques and the importance of utilizing protective equipment. Departments should also provide adequate shielding options for personnel to ensure that occupational radiation dose is kept as low as reasonably achievable.

Of all the reviewed literature, only three authors looked purely at dose to nurses during fluoroscopic procedures^39^, ^63^, ^64^ indicating that more studies are needed focussing on the occupational dose to nursing staff during x‐ray guided CV procedures.

## CONFLICT OF INTEREST

The authors declare no conflict of interest.
